# Enterotoxigenic *Bacteroides fragilis* infection exacerbates tumorigenesis in AOM/DSS mouse model

**DOI:** 10.7150/ijms.38371

**Published:** 2020-01-01

**Authors:** Soonjae Hwang, Chang Gun Lee, Minjeong Jo, Chan Oh Park, Sun-Yeong Gwon, Samnoh Hwang, Hye Chin Yi, So-Yeon Lee, Yong-Bin Eom, Baktiar Karim, Ki-Jong Rhee

**Affiliations:** 1 Department of Biomedical Laboratory Science, College of Health Sciences, Yonsei University at Wonju, Wonju, Gangwon-do 26493, Republic of Korea; 2 Cell Therapy and Tissue Engineering Center, Yonsei University Wonju College of Medicine, Wonju, 26426, Republic of Korea; 3 Department of Medical Science, College of Medical Sciences, Soonchunhyang University, Asan, Chungnam, 31538, Republic of Korea; 4 Department of Biomedical Laboratory Science, College of Medical Sciences, Soonchunhyang University, Asan, Chungnam, 31538, Republic of Korea; 5 Leidos Biomedical Research Inc, Frederick National Laboratory for Cancer Research, Frederick, MD, 21702, U.S.A.

**Keywords:** ETBF, colorectal cancer, inflammation, azoxymethane, dextran sulfate sodium

## Abstract

The azoxymethane (AOM)/dextran sulfate sodium (DSS) murine model is commonly used to study colitis-associated cancer. The human commensal bacterium, enterotoxigenic *Bacteroides fragilis* (ETBF) secretes the *Bacteroides fragilis* toxin (BFT) which is necessary and sufficient to cause colitis. We report that BALB/c mice infected with WT-ETBF and administered three cycles of AOM/DSS developed numerous, large-sized polyps predominantly in the colorectal region. In addition, AOM/DSS-treated BALB/c mice orally inoculated with wild-type nontoxigenic *Bacteroides fragilis* (WT-NTBF) overexpressing *bft* (rETBF) developed numerous polyps whereas mice infected with WT-NTBF overexpressing a biologically inactive *bft* (rNTBF) did not promote polyp formation. Unexpectedly, the combination of AOM+ETBF did not induce polyp formation whereas ETBF+DSS did induce polyp development in a subset of BALB/c mice. In conclusion, WT-ETBF promoted polyp development in AOM/DSS murine model with increased colitis in BALB/c mice. The model described herein provides an experimental platform for understanding ETBF-induced colonic tumorigenesis and studying colorectal cancer in wild-type mice.

## Introduction

Colorectal cancer is a leading cause of death in developed countries 1. Although genetic mutation is the main etiological factor, environmental factors greatly influence the pathogenesis of colorectal cancer 2, 3. Among these factors, inflammation plays a pivotal role in enhancing tumor survival and progression through activation pro-inflammatory cytokines 4. Numerous studies have highlighted the association between microbial-induced inflammation and cancer 5, 6.

Enterotoxigenic* Bacteroides fragilis* (ETBF) is a human colonic commensal associated with juvenile diarrhea as well as high-grade colorectal cancer 7, 8. The key virulence factor of ETBF is attributed to *B. fragilis* toxin (BFT), a secreted 20-kDa zinc-dependent metalloprotease toxin present as three isotypes (BFT-1, BFT-2, and BFT-3) 9. BFT induces E-cadherin cleavage in colonic epithelial cells *in vivo* resulting in increased intestinal permeability and subsequent colitis. BFT activates β-catenin, NF-κB and MAPK pathways, leading to enhanced cellular proliferation and secretion of IL-8 in HT29/C1 cells 10, 11. In animal models, BFT is sufficient and necessary to induce colitis in both mice and gerbils 12-14. Wild-type C57BL/6 mice harboring ETBF for 18 months exhibit chronic colitis but show no signs of polyps 12. However, ETBF infection promoted Th17-dependent colonic tumorigenesis in the APC^Min/+^ mouse model 15. APC^Min/+^ mice harbor a heterozygous germline mutation in the APC gene and is an animal model with spontaneous tumorigenesis for familial adenomatous polyposis (FAP). APC^Min/+^ mice are often used to assess anti-tumor drugs and nutritional risk factors, and are utilized to elucidate mechanisms of colonic tumorigenesis *in vivo*. Another often-used animal model for colorectal cancer is the co-administration of azoxymethane (AOM), genotoxic carcinogen and dextran sulfate sodium (DSS), a non-genotoxic sulfated polysaccharide 16. An advantage of the AOM/DSS system is the ease of manipulation and documented efficacy in wild-type mice strains (e.g., BALB/c and C57BL/6). Prolonged AOM/DSS treatment induces progression from adenomas to adenocarcinomas in a subset of mice, depending on the mouse strain and treatment conditions 17, 18. Furthermore, all polyps in AOM/DSS-treated mice form in the colon. In the current study, we assessed the tumorigenic potential of ETBF colonization in the AOM/DSS mouse model. We found that ETBF colonization in wild-type BALB/c mice administered with AOM/DSS resulted in a rapid development of a large number of polyps predominantly in the colorectal region. The protocol described herein may be readily implemented to study ETBF tumorigenesis in wild-type mice.

## Materials and Methods

### Bacterial strains

The wild-type ETBF strains used were as follows: *B. fragilis* VPI 13784 (*bft-1*), *B. fragilis* 86-5443-2-2 (*bft-2*), and *B. fragilis* Korea 570 (*bft-3*). The nontoxigenic wild-type *B. fragilis* (WT-NTBF) strain, *B. fragilis* NCTC 9343, lacks the expression of BFT. WT-NTBF recombinant strains overexpressing active BFT (rETBF; *bft-2*) and WT-NTBF overexpressing a biologically inactive mutated BFT (rNTBF; *bft-2* H352Y) were previously reported 12, 19. Both rETBF and rNTBF strains have a clindamycin-resistant gene on a plasmid. All wild-type *Bacteroides* strains used in this study are resistant to gentamicin. pFD340 is a plasmid vector conferring clindamycin resistance to transformed *Bacteroides* strains. Therefore, all strains used in this study are resistant to clindamycin either naturally or by introduction of pFD340. Bacterial strains were a generous gift from Cynthia Sears and Augusto Franco-Mora (Johns Hopkins University, USA).

### Mouse experiments

All animal housing and experimental procedures has been reviewed and approved by the Institutional Animal Care and Use Committee of Yonsei University at Wonju (YWC-151005-1, YWCI-201612-014-01) and Institutional Biosafety Committee of Yonsei University at Wonju (201612-P-014-01). All experiments were performed to conform to relevant guidelines and regulations under the Institutional Animal Care and Use Committee of Yonsei University at Wonju and Institutional Biosafety Committee of Yonsei University at Wonju. Eight-week-old female BALB/c mice (Raon Bio, Korea) received a single intraperitoneal injection of AOM (10 mg/kg). Two days later, mice were provided drinking water containing clindamycin (100 mg/L) and gentamicin (300 mg/L) for 5 days to promote colonization of *B. fragilis*. Mice were inoculated with bacteria and antibiotic-containing water was continued for additional 7 days. After 7 days of distilled water (DW), the first DSS cycle was initiated (5 days DSS, 16 days of DW) for a total of three cycles. DSS (36-50 kDa) was purchased from MP Biomedicals. AOM was obtained from Sigma-Aldrich. Bacteria were grown in brain heart infusion broth and adjusted to 1 × 10^9^ CFU/200 μL for mouse oral inoculations. Colonization of bacteria was monitored by serial dilution and plating of stool on brain heart infusion agar plates containing gentamicin (50 μg/mL) and clindamycin (6 μg/mL). Characteristic *B. fragilis* colonies were enumerated after anaerobic culture and shown as CFU/gram stool. All mice harbored the inoculated strains at 1 × 10^9^ CFU/gram stool during the 12-week period (data not shown).

### Distribution of polyp size

Unstained large intestine was examined by two investigators using a stereomicroscope to determine the polyp number and size. The length and width of the polyp were measured using a micro caliper. Polyp sized was calculated as length × width. Polyps were grouped as < 2 mm^2^, 2~4 mm^2^, and > 4 mm^2^. Results are shown as median polyp size.

### Histology

The large intestine (colon and cecum) was excised and fixed in 10% formalin. For microscopic examination, tissues were embedded in paraffin and sectioned (5 μm) with a rotary microtome. Slides were stained with hematoxylin, bluing buffer, eosin for 1 minute each, dehydrated using alcohol (95%, 100%, 100%, 100%) for 1 minute each and then rinsed in xylene for two times 1 minute each. Slides were photographed by optical microscopy (Leica, Germany) and rendered using Adobe Photoshop and Leica software.

### Quantitative reverse transcription polymerase chain reaction (qRT-PCR)

Frozen colonic tissues were homogenized using plastic homogenizer for 10 minutes. Total RNA was isolated using TRIzol reagent (Life Technologies, CLD, CA) according to the manufacturer's instructions. cDNA was synthesized using M-MLV Reverse Transcriptase kit (Life Technologies, CLD, CA, USA) and random primers. Detection of genes was performed using gene-specific primers in the presence of SYBR green. The qRT-PCR protocol was 95°C for 10 minutes, followed by 40 cycles of 95°C for 15 seconds, 55°C for 30 seconds, and 72°C for 1 minute. Data were normalized to *gapdh* expression.

### Multiplex cytokine bead array

Serum samples from mice were obtained via cardiac puncture and centrifuged. Serum was stored at -20°C until analysis. Serum levels of IL-17A and KC were determined by ELISA kit (R&D System, Minneapolis, MN, USA). Th17-associated cytokines were measured using multiplex cytokine magnetic bead array (Merck, Darmstadt, Germany) for simultaneous analysis of multiple cytokines.

### Statistics

All statistical analyses were performed using the Mann-Whitney test (GraphPad Prism). A value of *P* < 0.05 was regarded as significant.

## Results

### ETBF infection enhances colonic tumorigenesis in AOM/DSS model

**Figure 1:**
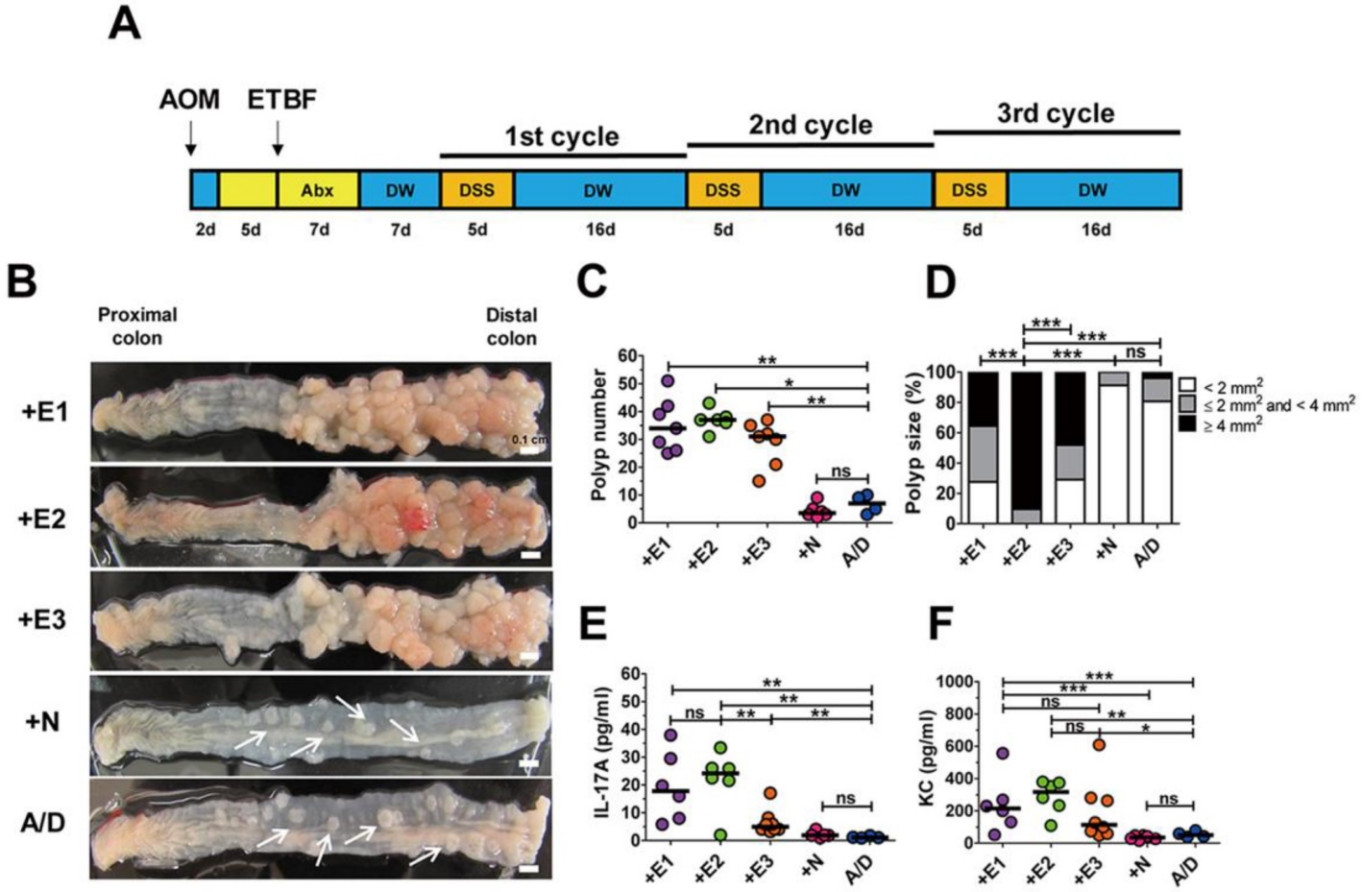
** ETBF infection enhances colonic tumorigenesis in AOM/DSS model. (A)** ETBF/AOM/DSS protocol. BALB/c mice were given a single intraperitoneal injection of AOM (10 mg/kg) and provided drinking water *ad libitum* containing clindamycin/gentamicin for 5 days. WT-ETBF strains (BFT-1, BFT-2 or BFT-3 isotype; 1 × 10^9^ CFU) were orally inoculated and the antibiotic cocktail continued for an additional 7 days. Seven days later, mice were subjected to three cycles of regular water (16 days per cycle) and 2% DSS (5 days per cycle) treatment. Total experimental period was 12 weeks. **(B)** Representative gross macroscopic image of the colon. A/D, AOM/DSS alone; +E1, AOM/DSS + WT-ETBF (*bft-1*); +E2, AOM/DSS + WT-ETBF (*bft-2*); +E3, AOM/DSS + WT-ETBF (*bft-3*); +N, AOM/DSS + WT-NTBF. White arrows indicate polyps. **(C)** Polyp number. **(D)** Polyp size distribution. **(E)** Serum IL-17 levels. **(F)** Serum KC levels. Each dot represents one mouse (n = 5-8 mouse per group). Horizontal bar, median. *P < 0.05, **P < 0.01, ***P < 0.001. ns, no statistical significance.

ETBF is categorized into three types depending on the secretion of one of the three BFT genes (i.e., *bft-1*, *bft-2*, and *bft-3*). We examined the tumorigenic potential of all three BFT types secreted by WT-ETBF strains in the AOM/DSS model. BALB/c mice were injected intraperitoneally with AOM (10 mg/kg) once and provided with drinking water containing clindamycin and gentamicin 2 days later for a total of 12 days (Fig. 1A). Thereafter, distilled water was provided for the duration of the experiment. WT-ETBF (1 × 10^9^ colony-forming units [CFU]) were orally inoculated once at day 7. At day 21, the 1^st^ DSS cycle (5 days of 2% DSS + 16 days of distilled water) was initiated with a total of three DSS cycles. Mice were euthanized at the end of the 3^rd^ DSS cycle and the colon examined macroscopically. We found that all three WT-ETBF strains dramatically increased polyp numbers in the AOM/DSS model (median polyp number = 32-48) compared to mice given only AOM/DSS (median polyp number = 7) (Fig. 1B,C). AOM/DSS mice infected with the nontoxigenic *B. fragilis* strain (WT-NTBF) that does not secrete BFT showed no increase in polyp number compared with the group given only AOM/DSS (Fig. 1C). The polyp size distribution data indicate that AOM/DSS mice **(*n = 6*)** infected with WT-ETBF exhibited larger-sized polyps compared to mice **(*n = 6*)** infected with WT-NTBF or uninfected AOM/DSS mice **(*n = 4*)** (Fig. 1D). In addition, the BFT-2 secreting WT-ETBF was more effective in enhancing polyp growth compared to BFT-1 **(*n = 7*)** and BFT-3 **(*n = 6*)** secreting WT-ETBF. These data collectively show that ETBF infection dramatically increased polyp number and size in AOM/DSS mice.

A positive correlation exists for elevated levels of the inflammatory cytokines IL-17 and IL-8 with inflammatory disorders 20, 21. To determine if these two inflammatory cytokines were elevated in WT-ETBF infected AOM/DSS mice, serum IL-17A and KC (murine functional homologue of human IL-8) levels were examined by ELISA. We found that both IL-17A and KC levels were elevated in WT-ETBF infected AOM/DSS mice compared to WT-NTBF infected AOM/DSS mice and AOM/DSS alone mice (Fig. 1E, F). Among the WT-ETBF infected groups, BFT-2 secreting WT-ETBF exhibited the highest levels of these two cytokines. This result suggests a positive correlation with the ability of WT-ETBF to induce IL-17A/KC and its ability to increase polyp numbers and size.

### ETBF infection elevates pro-tumorigenic inflammatory cytokine levels in the AOM/DSS model

**Figure 2:**
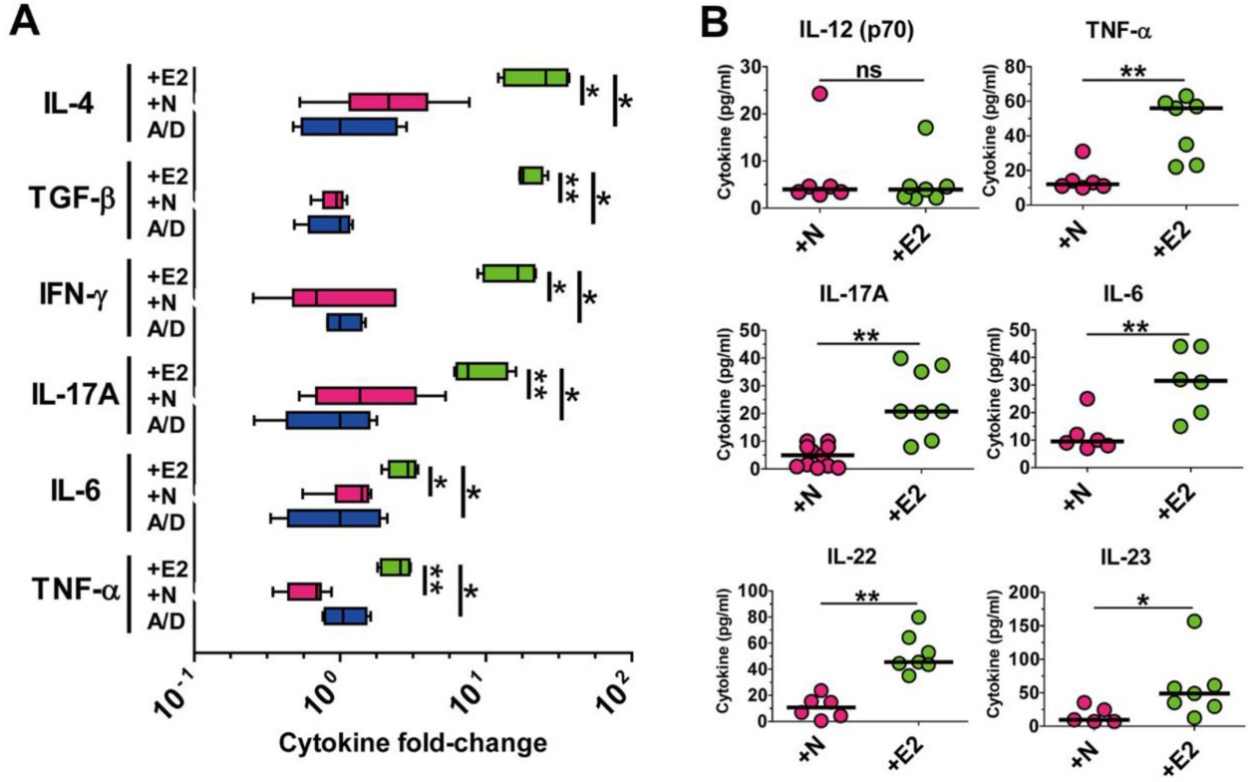
** ETBF infection elevates pro-tumorigenic inflammatory cytokine levels in the AOM/DSS model.** BALB/c mice were infected with WT-ETBF (*bft-2*) or WT-NTBF and subjected to the standard ETBF/AOM/DSS (2%) protocol for 12 weeks. A/D, AOM/DSS alone; +E2, WT-ETBF (*bft-2*) + AOM/DSS; +N, WT-NTBF + AOM/DSS. **(A)** qRT-PCR analysis of cytokine genes in colonic tissues. Box and whisker plot. **(B)** Multiplex cytokine bead array analysis of serum cytokines. Scatter plot. Each dot represents one mouse. Horizontal bar, median. *P < 0.05, **P < 0.01. ns, no statistical significance.

Both clinical and non-clinical studies have demonstrated that chronic intestinal inflammation and the accompanying inflammatory cytokines are conducive to colonic tumorigenesis 2, 4 . Wu et. al also showed that APC^Min/+^ mice infected with ETBF elaborated a myriad of inflammatory cytokines especially of the Th17-axis cytokines 15. Having observed that ETBF infection increased polyp formation in AOM/DSS-treated mice, we hypothesized that ETBF-infected AOM/DSS mice would also show increased levels of these cytokines. Cytokine analysis was performed by real-time polymerase chain reaction (PCR) of distal colonic tissues and cytokine bead array of serum cytokines. Results indicate that levels of all major inflammatory cytokines were significantly elevated in distal colonic tissues from WT-ETBF (*bft-2*) infected AOM/DSS mice compared with those in WT-NTBF infected AOM/DSS mice (Fig. 2A). Moreover, the Th17-axis cytokines IL-17A, IL-6, IL-22 and IL-23 were significantly elevated in serum of WT-ETBF infected AOM/DSS mice as compared with those in serum of WT-NTBF infected AOM/DSS mice (Fig. 2B). This data by PCR and cytokine bead array are statistically significant and consistent with the hypothesis that WT-ETBF promotes the Th17 immune response-mediated tumorigenesis in AOM/DSS murine model.

### Biologically active BFT is necessary for enhancing colonic tumorigenesis in the AOM/DSS model

**Figure 3:**
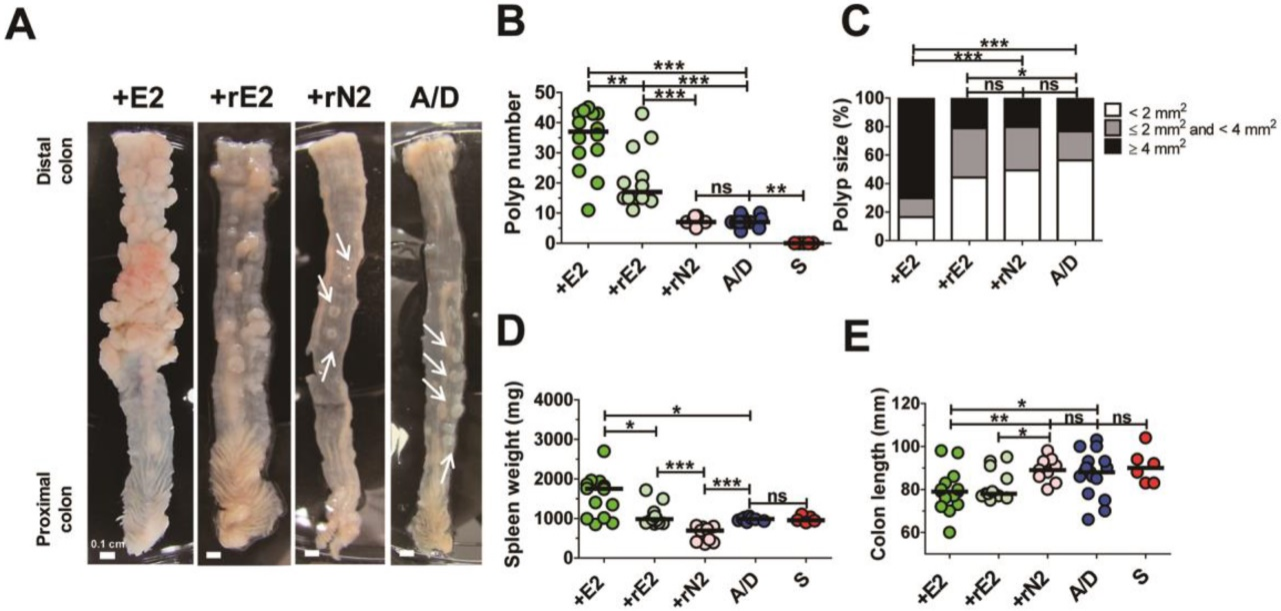
** Biologically active BFT is necessary for enhancing colonic tumorigenesis in the AOM/DSS model.** BALB/c mice were infected with WT-ETBF (*bft-2*), rETBF (*bft-2*) or rNTBF (*bft-2* H352Y) and subjected to the standard ETBF/AOM/DSS (2%) protocol for 12 weeks. +E2, WT-ETBF (*bft-2*) + AOM/DSS; +rE2, rETBF (*bft-2*) + AOM/DSS; rN2 (*bft-2* H352Y) + AOM/DSS; A/D, AOM/DSS alone. **(A)** Representative gross macroscopic image of the colon. White arrows indicate polyps. **(B)** Polyp number. S, sham control. **(C)** Polyp size distribution. **(D)** Spleen weight. **(E)** Colon length. Each dot represents one mouse (n = 9-12 mice per group). Horizontal bar, median. *P < 0.05, **P < 0.01, ***P < 0.001. ns, no statistical significance.

ETBF strain secretes the catalytically active BFT which is necessary and sufficient to cause colitis in infected mice and gerbils 12, 14. Therefore, we determined whether enhanced tumorigenesis in the ETBF/AOM/DSS model requires secretion of a biologically active BFT. AOM/DSS BALB/c mice were inoculated with a WT-ETBF (*bft-2*), nontoxigenic *B. fragilis* strain harboring a plasmid containing the active BFT-2 gene (rETBF), or nontoxigenic *B. fragilis* secreting catalytically inactive BFT (rNTBF). We found that mice inoculated with WT-ETBF (*bft-2*) **(*n* = 14)** and rETBF **(*n* = 12)** showed increased polyps, whereas those inoculated with rNTBF **(*n* = 9)** developed polyps comparable to those observed in mice administered AOM/DSS alone **(*n* = 14)** (Fig. 3A,B). Polyp size was also larger in WT-ETBF (*bft-2*) mice compared to rETBF infected mice (Fig 3C). However, rETBF-infected AOM/DSS mice developed fewer polyps (median = 16 polyps per mouse) compared to WT-ETBF (*bft-2*) infected AOM/DSS mice (median = 37 polyps per mouse). This led us to consider whether ETBF colonization differed among these two groups. However, periodic quantification of bacterial burden in the stool of mice found no differences (median = 2.6 × 10^9^ CFU/gram stool in WT-ETBF mice versus 3.5 × 10^9^ CFU/gram stool in rETBF mice). Increased spleen weight and decreased colon length, all indicative of enhanced colonic inflammation was observed in WT-ETBF and rETBF mice as compared with those in rNTBF mice (Fig. 3D,E). Serum IL-17A levels were consistent with this result (Supplemental Fig. 1). Collectively, these data suggest that a biologically active BFT is necessary to promote tumorigenesis in the AOM/DSS model. However, WT-ETBF has a greater tumorigenic potential compared to rETBF, suggesting additional factors provided by WT-ETBF may be necessary to fully enhance tumorigenesis. Paradoxically, rNTBF secreting the biologically inactive form of BFT-2 significantly decreased spleen weight in the AOM/ DSS model compared to AOM/DSS alone (Fig. 3D).

### The combination of ETBF+DSS can induce colonic tumorigenesis in BALB/c mice

**Figure 4:**
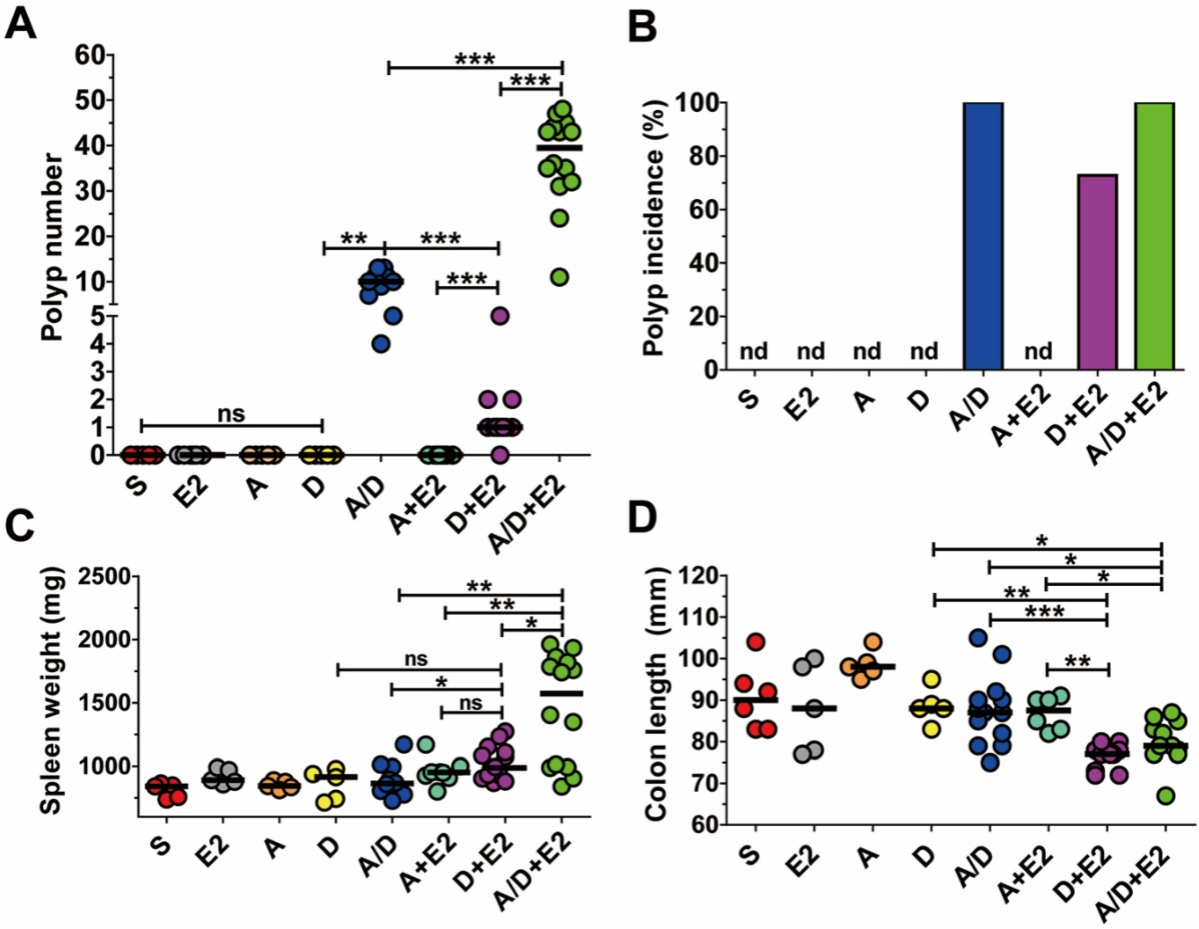
** The combination of ETBF+DSS can induce colonic tumorigenesis in BALB/c mice.** BALB/c mice were infected with WT-ETBF (*bft-2*) and subjected to the standard ETBF/AOM/DSS (2%) protocol for 12 weeks. Different combinations of reagents and/or bacteria were omitted. S, sham control with no ETBF, AOM, and DSS; E2, infected with WT-ETBF (*bft-2*) alone; A, AOM alone; D, DSS alone; A/D, AOM+DSS; A+E2, AOM+WT-ETBF (*bft-2*); D+E2, DSS+WT-ETBF (*bft-2*); A/D+E2, AOM+DSS+WT-ETBF (*bft-2*). **(A)** Polyp number. **(B)** Polyp incidence. **(C)** Spleen weight. **(D)** Colon length. Each dot represents one mouse (n = 9-15 mice per group). Horizontal bar, median. *P < 0.05, **P < 0.01, ***P < 0.001. ns, no statistical significance. nd, not detected.

In the AOM/DSS model, tumorigenesis is the result of DNA damage initiated by AOM and inflammation caused by DSS. Several studies have demonstrated that ETBF induces colonic inflammation and a more recent study suggested that BFT may cause DNA damage 22. As AOM/DSS-treated mice infected with ETBF exhibited increased polyp number, we determined whether ETBF acted as an initiator of polyp formation and/or enhancer of inflammation. BALB/c mice were administered AOM+ETBF or DSS+ETBF and examined for polyp formation. While AOM+ETBF-treated mice **(*n* = 9)** failed to induce polyp formation, ETBF+DSS-treated mice **(*n* = 11)** developed colonic polyps (median = 1 per mouse), with a polyp incidence of 70% (Fig. 4A,B). The tumorigenic ability of DSS+ETBF is not due to enhanced inflammation because ETBF+DSS did not increase spleen weight nor decrease colon length, both indirect parameters of inflammation, compared to AOM+ETBF mice (Fig. 4C,D). Consistently, IL-17 levels in serum were comparable between AOM+ETBF mice and DSS+ETBF mice (Supplemental Fig. 2). Finally, we considered the possibility that ETBF alone promote tumorigenesis in BALB/c mice. However, BALB/c mice infected with ETBF for up to 14 months developed no polyps (data not shown).

## Discussion

The primary objective of the present study was to determine the effects of ETBF infection tumorigenesis using AOM/DSS model. AOM/DSS is the most utilized murine model to study colitis-associated cancer. AOM/DSS model has also been used to assess anti-tumor effects of numerous anti-cancer drugs 23. The main advantage of this model is the usage of wild-type mice and hence the lack of maintenance of genetically defined mice such as the APC^Min/+^ mice. In addition, AOM and DSS are chemicals that are commercially available. Thus, the AOM/DSS model is readily implemented by small-sized laboratories. An often cited drawback to the AOM/DSS model is the usage of artificial chemicals (i.e., AOM and DSS) thus lacking physiologic relevance. Regardless, the AOM/DSS models has been used to understand key mechanisms of colon tumorigenesis. Herein, we show that wild-type BALB/c mice infected with ETBF and administered AOM/DSS developed numerous colorectal polyps within a 12-week period.

All three BFT isotypes induced polyp numbers equally well with the WT-ETBF (BFT-2) inducing larger-sized polyps. The induction of polys was due to secretion of biologically active BFT as the rNTBF strain did not induce polyps above the AOM/DSS group. The WT-ETBF (BFT-2) was more effective in inducing colon tumors and inflammation compared to rETBF (BFT-2). This result suggests that additional virulence factor(s) produced by WT-ETBF (BFT-2) further promote tumorigenesis. Consistent with this observation, 100% of germ-free 129S6/SvEv mice monoassociated with WT-ETBF (BFT-2) exhibited 100% mortality within 3 days but no mortality for germ-free mice monoassociated with rETBF (BFT-2) although colonic inflammation was comparable in mice infected with WT-ETBF (BFT-2) and rETBF (BFT-2) 12. We considered the possibility that rETBF (BFT-2) lost the plasmid containing the BFT-2 gene during the 12-week AOM/DSS treatment. However, periodic assessment of rETBF colonization using selective bacterial plates containing clindamycin showed that the colonization of rETBF was maintained a high level comparable to the three WT-ETBF strains. In addition, we examined the number of ETBF in feces from AOM/DSS-treated mice administered WT-ETBF (BFT-2), rETBF (BFT-2) and rNTBF (BFT-2 H352Y) but found comparable numbers among the groups (data not shown). Interestingly, Ly et al recently conducted a similar experiment, wherein AOM/DSS mice orally administered with purified recombinant BFT showed tumor suppression 24. The mechanistic explanation for this observation is unclear, but we suggest that the oral administration of purified recombinant BFT resulted in denaturation of the BFT during transit through the low pH environment of the stomach, rendering it biologically inactive. Contrary to our expectations, another unexpected result in the current study was that mice given AOM+ETBF did not induce polyp formation whereas mice given ETBF+DSS did induce polyp formation (median = 1, range 0-5). We expected that ETBF would acts as a colitis-inducing factor in lieu of DSS and thus AOM+ETBF would induce polyps. The fact that ETBF+DSS induced polyps, albeit to the lesser degree compared to AOM/DSS/ETBF groups, suggest that ETBF provided a tumor-initiating factor 22.

In order to investigate mechanistic link, we analyzed levels of serum IL-17A in mice given AOM+ETBF or ETBF+DSS, and found that there was no statistical difference in serum IL-17A levels between the AOM+ETBF and DSS+ETBF groups (Supplemental Fig. 1). Furthermore, comparable spleen weight between mice given AOM+ETBF and ETBF+DSS supports the speculation that polyp formation in mice given DSS+ETBF is not primarily resulted from increased colon inflammation. BFT induces spermine oxidase (SMO)-mediated DNA damage in T84 human colorectal cancer cells *in vitro* and ETBF infection increases SMO expression in colon epithelial cells in mice 22. Furthermore, ETBF infection-mediated inflammation promotes polyps in APC^Min/+^ mice 15. However, it was reported that inflammation by ETBF is not sufficient to induce polyps in wild-type C57BL/6 mice even at 18 months of continuous infection. 12. DSS is known to damage epithelial integrity thereby increasing gut permeability 25. In current study, repeated DSS exposure might amplify BFT-induced SMO-mediated DNA damage and inflammatory response by ETBF, which might be expected to be sufficient to induce DNA damage to initiate polyp formation.

In summary, we report that ETBF infection increases tumorigenesis in AOM/DSS-treated wild-type mice via enhancing colon inflammation, which is dependent on BFT activity. DSS treatment does not impact ETBF colonization *in vivo*. Co-treatment of ETBF and DSS induces synergistic effects of polyp formation in wild-type mice. ETBF-mediated tumorigenesis model could be used for investigating mechanistic factors related with pathogenesis of colorectal cancer promoted by ETBF.

## Supplementary Material

Supplementary figures.

## References

[1] Center MM, Jemal A, Smith RA, Ward E (2009). Worldwide variations in colorectal cancer. CA Cancer J Clin.

[2] Eaden JA, Abrams KR, Mayberry JF (2001). The risk of colorectal cancer in ulcerative colitis: a meta-analysis. Gut.

[3] Fearon ER (2011). Molecular genetics of colorectal cancer. Annu Rev Pathol.

[4] Grivennikov S, Karin E, Terzic J, Mucida D, Yu GY, Vallabhapurapu S (2009). IL-6 and Stat3 are required for survival of intestinal epithelial cells and development of colitis-associated cancer. Cancer Cell.

[5] Karin M, Lawrence T, Nizet V (2006). Innate immunity gone awry: linking microbial infections to chronic inflammation and cancer. Cell.

[6] Mariathasan S, Monack DM (2007). Inflammasome adaptors and sensors: intracellular regulators of infection and inflammation. Nat Rev Immunol.

[7] Boleij A, Hechenbleikner EM, Goodwin AC, Badani R, Stein EM, Lazarev MG (2015). The *Bacteroides fragilis* toxin gene is prevalent in the colon mucosa of colorectal cancer patients. Clin Infect Dis.

[8] Sears CL, Islam S, Saha A, Arjumand M, Alam NH, Faruque AS (2008). Association of enterotoxigenic *Bacteroides fragilis* infection with inflammatory diarrhea. Clin Infect Dis.

[9] Sears CL (2001). The toxins of *Bacteroides fragilis*. Toxicon.

[10] Hwang S, Gwon SY, Kim MS, Lee S, Rhee KJ (2013). *Bacteroides fragilis* toxin induces IL-8 secretion in HT29/C1 cells through disruption of E-cadherin junctions. Immune Netw.

[11] Wu S, Powell J, Mathioudakis N, Kane S, Fernandez E, Sears CL (2004). *Bacteroides fragilis* enterotoxin induces intestinal epithelial cell secretion of interleukin-8 through mitogen-activated protein kinases and a tyrosine kinase-regulated nuclear factor-κB pathway. Infect Immun.

[12] Rhee K-J, Wu S, Wu X, Huso DL, Karim B, Franco AA (2009). Induction of persistent colitis by a human commensal, enterotoxigenic *Bacteroides fragilis*, in wild-type C57BL/6 mice. Infect Immun.

[13] Hwang S, Lee MH, Gwon SY, Lee S, Jung D, Rhee KJ (2013). Analysis of inflammatory cytokines from the cecum and proximal colon of mice infected with enterotoxigenic *Bacteroides fragilis*. J Exp Biomed Sci.

[14] Yim S, Gwon SY, Hwang S, Kim NH, Jung BD, Rhee KJ (2013). Enterotoxigenic *Bacteroides fragilis* causes lethal colitis in Mongolian gerbils. Anaerobe.

[15] Wu S, Rhee KJ, Albesiano E, Rabizadeh S, Wu X, Yen HR (2009). A human colonic commensal promotes colon tumorigenesis via activation of T helper type 17 T cell responses. Nat Med.

[16] Tanaka T, Kohno H, Suzuki R, Yamada Y, Sugie S, Mori H (2003). A novel inflammation-related mouse colon carcinogenesis model induced by azoxymethane and dextran sodium sulfate. Cancer Sci.

[17] Suzuki R, Kohno H, Sugie S, Nakagama H, Tanaka T (2006). Strain differences in the susceptibility to azoxymethane and dextran sodium sulfate-induced colon carcinogenesis in mice. Carcinogenesis.

[18] Suzuki R, Kohno H, Sugie S, Tanaka T (2005). Dose-dependent promoting effect of dextran sodium sulfate on mouse colon carcinogenesis initiated with azoxymethane. Histol Histopathol.

[19] Wu S, Rhee KJ, Zhang M, Franco A, Sears CL (2007). *Bacteroides fragilis* toxin stimulates intestinal epithelial cell shedding and γ-secretase-dependent E-cadherin cleavage. J Cell Sci.

[20] Karabulut S, Usul Afsar C, Karabulut M, Kilic L, Alis H, Kones O (2016). Clinical significance of serum interleukin-17 levels in colorectal cancer patients. J BUON.

[21] Zhu Q, Man SM, Gurung P, Liu Z, Vogel P, Lamkanfi M (2014). STING mediates protection against colorectal tumorigenesis by governing the magnitude of intestinal inflammation. J Immunol.

[22] Goodwin AC, Destefano Shields CE, Wu S, Huso DL, Wu X, Murray-Stewart TR (2011). Polyamine catabolism contributes to enterotoxigenic *Bacteroides fragilis*-induced colon tumorigenesis. Proc Natl Acad Sci U S A.

[23] De Robertis M, Massi E, Poeta ML, Carotti S, Morini S, Cecchetelli L (2011). The AOM/DSS murine model for the study of colon carcinogenesis: From pathways to diagnosis and therapy studies. J Carcinog.

[24] Lv Y, Ye T, Wang HP, Zhao JY, Chen WJ, Wang X (2017). Suppression of colorectal tumorigenesis by recombinant *Bacteroides fragilis* enterotoxin-2 *in vivo*. World J Gastroenterol.

[25] Lee JS, Tato CM, Joyce-Shaikh B, Gulen MF, Cayatte C, Chen Y (2015). Interleukin-23-independent IL-17 production regulates intestinal epithelial permeability. Immunity.

