# Supplementary material for: Enterotoxigenic Bacteroides fragilis infection exacerbates tumorigenesis in AOM/DSS mouse model
Source: Int J Med Sci. 2020 Jan 1;17(2):145–52. doi: 10.7150/ijms.38371 (PMC6990882; doi:10.7150/ijms.38371)

Supplementary figure legend

Supplementary figure 1. Serum IL-17A levels in AOM+ETBF mice and DSS+ETBF mice. BALB/c mice were injected with AOM and infected with WT-ETBF (*bft-2*) or administered DSS (2%) for 12 weeks. E2, infected with WT-ETBF (*bft-2*) alone; A, AOM alone; A+E2, AOM+WT-ETBF (*bft-2*); D+E2, DSS+WT-ETBF (*bft-2*). Each dot represents one mouse (n = 5–8 mouse per group). Horizontal bar, median. *$P < 0.05$, ns, no statistical significance.

Supplementary figure 2. Active BFT increases serum IL-17A levels in AOM/DSS mice. BALB/c mice were infected with WT-ETBF (*bft-2*), rETBF (*bft-2*) or rNTBF (*bft-2* H352Y) and subjected to the standard ETBF/AOM/DSS (2%) protocol for 12 weeks. +E2, WT-ETBF (*bft-2*) + AOM/DSS; +rNT, rNTBF (*bft-2* H352Y) + AOM/DSS; rET, rETBF (*bft-2*) + AOM/DSS; A/D, AOM/DSS alone. Each dot represents one mouse (n = 6–11 mouse per group). Horizontal bar, median. **$P < 0.01$, ***$P < 0.001$. ns, no statistical significance.

Supplementary figure 1

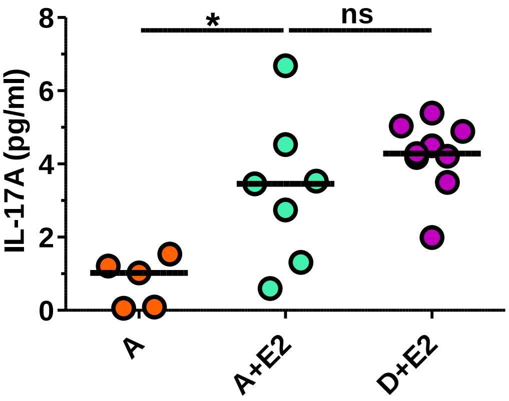

Supplementary figure 2

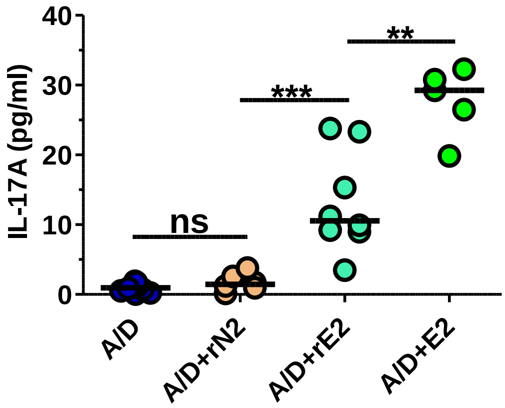

Supplement: Supplementary file 1 — Supplementary figures. [file ijmsv17p0145s1.pdf]
